# Addendum: Transmission of MPXV from fire-footed rope squirrels to sooty mangabeys

**DOI:** 10.1038/s41586-026-10767-2

**Published:** 2026-06-24

**Authors:** Carme Riutord-Fe, Jasmin Schlotterbeck, Lorenzo Lagostina, Leonce Kouadio, Harriet R. Herridge, Moritz J. S. Jochum, Nea Yves Noma, Ane López-Morales, Donata Hoffmann, Sten Calvelage, Hjalmar Kühl, Alexander Mielke, Catherine Crockford, Liran Samuni, Roman M. Wittig, Martin Beer, Sery Gonedelé-Bi, Jan F. Gogarten, Sébastien Calvignac-Spencer, Ariane Düx, Livia V. Patrono, Fabian H. Leendertz

**Affiliations:** 1 Department of Ecology and Emergence of Zoonotic Diseases, Helmholtz Institute for One Health (HIOH), Helmholtz Centre for Infection Research (HZI), Greifswald, Germany; 2 Taï Chimpanzee Project, Centre Suisse de Recherches Scientifiques en Côte d’Ivoire, Abidjan, Côte d’Ivoire; 3 Université Peleforo Gon Coulibaly Korhogo, Korhogo, Côte d’Ivoire; 4 Senckenberg Museum for Natural History Görlitz, Senckenberg—Member of the Leibniz Association, Görlitz, Germany; 5 Institute of Diagnostic Virology, Friedrich Loeffler Institute, Riems, Germany; 6 International Institute Zittau, Technische Universität Dresden, Zittau, Germany; 7 Centre for Brain and Behaviour, Department of Psychology, Queen Mary University of London, London, UK; 8 Ape Social Mind Lab, Institute of Cognitive Sciences, CNRS University of Lyon, Lyon, France; 9 Cooperative Evolution Lab, German Primate Center, Göttingen, Germany; 10 Laboratoire de Biotechnologie, Agriculture et Valorisation des Ressources Biologiques, UFR Biosciences, Université Félix Houphouët-Boigny d’Abidjan-Cocody, Abidjan, Côte d’Ivoire; 11 Evolutionary Community Ecology Research Group, Helmholtz Institute for One Health (HIOH), Helmholtz Centre for Infection Research (HZI), Greifswald, Germany; 12 University of Greifswald, Greifswald, Germany; 13 Department of Pathogen Evolution, Helmholtz Institute for One Health (HIOH), Helmholtz Centre for Infection Research (HZI), Greifswald, Germany; 14 University Medicine Greifswald, Greifswald, Germany

**Keywords:** Viral transmission, Viral infection, Ecological epidemiology, Infectious-disease diagnostics, Pathogens

Addendum to: *Nature* 10.1038/s41586-025-10086-y Published online 11 February 2026

In our *Nature* paper we identified transmission of MPXV from a fire-footed rope squirrel (*F. pyrropus*) to sooty mangabeys. A recent study suggested the elevation of West African populations of fire-footed rope squirrel to a separate species under the name *F. leucostigma* (ref. ^1^). We agree with this proposal and report that a mitogenomic phylogeny places the fire-footed rope squirrel in our study in the new taxon. This taxonomic revision has not yet been broadly adopted by major reference databases, so we retained the currently predominant nomenclature (a single species name for all populations: *F. pyrropus*) in our article.
